# Neurogenic Stem Cell Niche in the Auditory Thalamus: In Vitro Evidence of Neural Stem Cells in the Rat Medial Geniculate Body

**DOI:** 10.3390/life13051188

**Published:** 2023-05-15

**Authors:** Jonas Engert, Bjoern Spahn, Linda Bieniussa, Rudolf Hagen, Kristen Rak, Johannes Voelker

**Affiliations:** Department of Otorhinolaryngology, Plastic, Aesthetic and Reconstructive Head and Neck Surgery, University Hospital Wuerzburg, Josef-Schneider-Strasse 11, 97080 Wuerzburg, Germany; spahn_b@ukw.de (B.S.); bieniussa_l@ukw.de (L.B.); hagen_r@ukw.de (R.H.); rak_k@ukw.de (K.R.); voelker_j@ukw.de (J.V.)

**Keywords:** neurosphere, auditory pathway, neural stem cell potential

## Abstract

The medial geniculate body (MGB) is a nucleus of the diencephalon representing a relevant segment of the auditory pathway and is part of the metathalamus. It receives afferent information via the inferior brachium of the inferior colliculus and transmits efferent fibers via acoustic radiations to the auditory cortex. Neural stem cells (NSCs) have been detected in certain areas along the auditory pathway. They are of great importance as the induction of an adult stem cell niche might open a regenerative approach to a causal treatment of hearing disorders. Up to now, the existence of NSCs in the MGB has not been determined. Therefore, this study investigated whether the MGB has a neural stem cell potential. For this purpose, cells were extracted from the MGB of PND 8 Sprague-Dawley rats and cultured in a free-floating cell culture assay, which showed mitotic activity and positive staining for stem cell and progenitor markers. In differentiation assays, the markers β-III-tubulin, GFAP, and MBP demonstrated the capacity of single cells to differentiate into neuronal and glial cells. In conclusion, cells from the MGB exhibited the cardinal features of NSCs: self-renewal, the formation of progenitor cells, and differentiation into all neuronal lineage cells. These findings may contribute to a better understanding of the development of the auditory pathway.

## 1. Introduction

Neurogenesis describes the process of the formation of new neurons from progenitor and stem cells [1]. The key features of neural stem cells (NSCs) are their ability to self-renew by mitosis and the potential to differentiate into neural progenitor cells and all neuronal lineage cells, including neurons, astrocytes, and oligodendrocytes [2]. NSCs are particularly promising to regenerative medicine as they may provide an endogenous therapeutic approach for damaged neuronal tissue [3]. In recent years, postnatal and adult neurogenesis has been described primarily in the dentate gyrus of the hippocampus [4]. Another neural stem cell niche was detected in the subventricular zone [5]. Outside these primary centers, neurogenic stem cell potential has been demonstrated in other nuclear areas: the neocortex, striatum, spinal cord, dorsal vagal complex, and optic nerve [6,7,8,9].

Recently, NSCs have also been described in the auditory system. For the first time in the inner ear, NSCs were detected in the utricle of the vestibular organ [10]. In the cochlea, neurogenic stem cells have been detected in the spiral ganglion [11]. Along the central auditory pathway, NSCs have been described in the rat and mouse cochlear nucleus (CN). The neurogenic stem cell potential of the CN was detected up to the adult stage [12]. In the midbrain, NSCs were detected in the rat inferior colliculus (IC) [13]. The mouse auditory cortex exhibits neural stem cell capacity into the adult stage [14]. In these studies, NSCs were analyzed indirectly by demonstrating their cardinal abilities. The cells showed the ability to undergo mitotic self-renewal, as evidenced by their ability to form neurospheres across multiple passages. In addition, immunocytological assays demonstrated that these cells differentiate into progenitor cells and all cell types of the neuroectodermal cell lineage, including neurons, astrocytes, and oligodendrocytes.

The medial geniculate body (MGB) is part of the auditory thalamus and represents the thalamic relay between the IC and the auditory cortex [15]. The MGB lies on the posterolateral surface of the thalamus as a rounded elevation. Adjacent, the MGB is ventral to the superior colliculus and medial to the hippocampus [15]. It marks the rostral pole of the brachium of the IC. The MGB is divided into three subnuclei: ventral, medial, and dorsal [16]. The ventral subnucleus projects to the primary auditory cortex and receives most of its anatomical input from the central nucleus of the IC, whereas the dorsal nucleus projects to the auditory association cortex. The medial nucleus appears to be functionally responsible for perceiving the relative intensity and duration of a sound [17].

As described, NSCs have already been detected in mammals in various nuclear areas, especially along the auditory pathway in early postnatal and adult animals. It is unknown whether the MGB has neurogenic stem cell potential. Therefore, this study aimed to examine cells of the MGB for the properties of NCCs in vitro and to analyze them for their ability to self-renew, proliferate and differentiate into progenitor cells, neurons, and neuroglial cells. Therefore, the study of the cells of the MGB will contribute to a better understanding of the developmental features of the auditory pathway.

## 2. Materials and Methods

### 2.1. Animal Preparation

**Figure 1:**
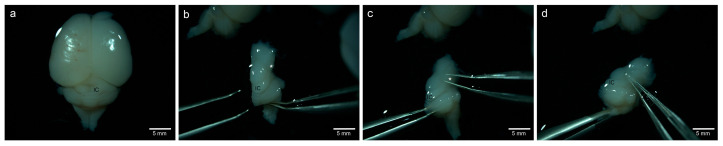
Preparation of the MGB of a PND 8 rat. (**a**) Dorsal view of the brainstem, cerebellum, midbrain, and cerebrum after exposure of meninges and supplying blood vessels. (**b**) Representation of the corpora quadrigemina with the IC and the diencephalon after removal of the cerebral hemispheres and vertical dissection in the midline. Tracing of the brachium colliculi inferioris and visualization of the MGB (*), which is the most caudally located extension laterally alongside the mesencephalon. (**c**) Identification of the IC and its brachium colliculi inferioris. Representation of the MGB (*). (**d**) Blunt dissection of the MGB (*).

Postnatal day (PND) 8 Sprague-Dawley rats (Charles River^®^, Wilmington, MA, USA) were euthanized by cervical dislocation and decapitation. The skull was opened midsagittally, and its bony parts were removed. After the cranial nerves were dissected, the brain with its brainstem was removed from the skull base and transferred into Neurobasal^®^ medium (Thermo-Fisher Scientific^®^, Grand Island, NE, USA) at room temperature. Using a stereomicroscope (OPMI1, Zeiss^®^, Oberkochen, Germany), lamina tecti with the superior and inferior colliculus was identified, and a blunt preparation and elevation of the cerebrum was performed to expose the diencephalon. For further orientation, the Brachium Colliculi Inferioris was exposed, starting from the lateral edge of the IC and proceeding to the MGB. After the identification of the MGB, the blunt dissection of the MGB was performed (Figure 1). The tissue was freed from meningeal tissue and blood vessels. The isolated tissues were transferred either into sterile DPBS solution or into Neurobasal^®^ medium at room temperature for further processing. All procedures were performed under antiseptic conditions.

All experiments were conducted according to the national guidelines for the care and use of laboratory animals (§8) and carried out exclusively as organ removal. Removing organs from the animal after the sacrifice is, as per §6 Abs. 1 No. 4 (German Animal Welfare Act), subject to a notification requirement but has not been and cannot be approved as an animal experiment. 

The number of sacrificed animals per species per year has to be given to the local authorities. Accordingly, nine sacrificed Sprague Dawley rats were reported to the “Regierung of Unterfranken” (Government Lower Franconia). 

### 2.2. Neurosphere Assay, NSC Medium and Passaging

The removed organ was transferred to Accutase (Gibco^®^, Thermo Fischer Scientific^®^, Grand Island, NE, USA) after preparation. Enzymatic dissociation was performed in a ThermoMixer^®^ (Eppendorf^®^, Hamburg, Germany) at 37 °C and 500 rpm, and the solution was triturated every 10 min using a 500-μL pipette. This was done to form an emulsion that was macroscopically free of tissue fragments. After dissolution, cells were centrifuged at 1000 rpm for 5 min (Centrifuge 5810, Eppendorf^®^, Hamburg, Germany), and the resulting pellet was resuspended in Neurobasal^®^ medium. Cell number was determined using an improved Neubauer hemocytometer (ZK06, Hartenstein^®^, Würzburg, Germany). Cell number was determined on a 10-μL sample mixed with 10 μL trypan blue (Thermo Fischer Scientific^®^, Grand Island, NE, USA), allowing quantification of vital cells. Free-swimming cell cultures were cultured in hydrophobic cell culture flasks (CELLLSTAR^®^, filter top, 25 cm^2^, Greiner^®^ Bio-One, Monroe, NC, USA) at 37 °C and 5% CO_2_ in a medium designated as NSC medium. This NSC medium consisted of serum-free Neurobasal^®^ medium, 1% GlutaMAX^®^ (Gibco^®^, Thermo Fischer Scientific^®^, Grand Island, NE, USA), 2% B27^®^ supplement without retinoic acid (Gibco^®^, Thermo Fischer Scientific^®^, Grand Island, NE, USA), and 1% penicillin/streptomycin (Gibco^®^, Thermo Fischer Scientific^®^, Grand Island, NE, USA). Recombinant murine growth factors EGF and bFGF/FGF-2 (PeproTech^®^, Thermo Fischer Scientific^®^, Grand Island, NE, USA) were added to these cultures at a final concentration of 10 ng/mL. The initial volume within a cell culture was 4 mL. Every four days, 2 mL of fresh NSC medium was added. The amount of primary forming cell spheres was assessed after 30 days using an inverted microscope (Leica^®^ DMI 4000B and DMI-8, Wetzlar, Germany) at 5× magnification. Subsequently, the calculation was performed in relation to 100,000 cultured cells per MGB. Dissociation of neurospheres for passaging was performed mechanically, and single cells were subsequently centrifuged at 1000 rpm for 5 min. Transfer to 4 mL of fresh NSC medium and cultivation for 30 days at 37 °C/5% CO_2_ in 50 mL / 25 cm^2^ Filter Top cell culture flasks (CELLLSTAR^®^, Filter Top, 25 cm^2^, Greiner^®^ Bio-One, Monroe, NC, USA) was performed. The absolute number of viable cells was determined before each passaging step, as mentioned above.

### 2.3. Plating of Neurospheres

The sampling of the neurospheres formed in the cell cultures was performed using a 5-mL automatic pipette (Multipette plus, Eppendorf^®^, Hamburg, Germany) and the neurospheres were applied to glass coverslips previously coated with poly-D-lysine (100 μg/mL, Serva Electrophoresis^®^, Thermo Fischer Scientific^®^, Grand Island, NE, USA) and laminin-1 (10 μg/mL, BD Biosciences^®^, Bergen County, NJ, USA). Per coverslip, 100 μL of NSC medium was added to the respective 4-well dishes (Greiner^®^ Bio-One^®^, Monroe, NC, USA), and the neurospheres were cultured for 24 h at 37 °C/5% CO_2_.

### 2.4. Plating of Single Cells, Analysis of Cell Division and Cell Differentiation

For single-cell preparation, the suspension with the neurospheres formed was taken and centrifuged at 1000 rpm for 5 min. The cell pellet was then rinsed once with DPBS to remove residual NSC medium. After aspiration of the PBS buffer solution, Accutase^®^ was added for cell dissociation. Incubation was performed for 15 min at 37 °C and 500 rpm in a Thermo-Mixer^®^ (Eppendorf^®^, Hamburg, Germany), and trituation of the cell suspension was performed every five minutes using a 200-μL pipette. Cells were subsequently centrifuged at 1000 rpm for 5 min, and the pellet was transferred to an NSC medium for the passage of cultures and recovery of secondary neurospheres. The number of cells was counted in the specific samples using an improved Neubauer^®^ hemocytometer. The number of viable cells was determined by adding trypan blue solution (0.4%; #93595—Sigma-Aldrich^®^, Merck^®^, St. Louis, MO, USA) to the measurement chamber. Single cells obtained in this manner were transferred as part of the suspension to a differentiation medium (DIF medium) consisting of Neurobasal^®^, GlutaMAX^®^, and B27 with retinoic acid. Cells were plated on glass coverslips coated with laminin (1:100 in PBS 0.05 M) and poly-d-lysine (PDL; 1:100 in 0.05 M PBS) at a density of 100 cells/mm^2^ (8000 cells/coverglass) and cultured in 4-well dishes at 37 °C/5% CO_2_ for eight days. The DIF medium was changed every two days.

### 2.5. Fixation and Immunocytochemistry

Single cells were fixed on glass coverslips with a 4% paraformaldehyde solution (PFA in 0.1 M NaPP) for 30 min, followed by acetone for 5 min. Blocking of non-specific binding sites was performed with a solution of 10% bovine serum albumin (BSA, A9418 Sigma-Aldrich^®^, Merck^®^, St. Louis, MO, USA) in 0.1 M PBS buffer solution (Sigma-Aldrich^®^, Merck^®^, St. Louis, MO, USA). Incubation with the following primary antibodies were performed for immunocytochemical assays at 5 °C for 12 h in 1% BSA solution and 0.1 M PBS buffer: mouse monoclonal antibody against ATOH1 (1:1000; Ab27667-Abcam^®^, Waltham, MA, USA), mouse monoclonal against β-tubulin (1:1000; #TS293—Sigma-Aldrich^®^, Merck^®^, St. Louis, MO, USA), mouse monoclonal versus β-III-tubulin (1:1000; #Ab7751—Abcam^®^, Waltham, MA, USA), rabbit polyclonal versus β-III-tubulin (1:2000; #Ab18207—Abcam^®^, Waltham, MA, USA), rabbit polyclonal against double cortin (DCX) (1:1000; #Ab18723—Abcam^®^, Waltham, MA, USA), mouse monoclonal protein against glial fibrillary acidic protein (GFAP) (1:1000; #MAB360—Merck Millipore^®^, Billerica, MA, USA), rabbit polyclonal against myelin basic protein (MBP) (1:800; #M3821—Sigma-Aldrich^®^, Merck^®^, St. Louis, MO, USA), rabbit polyclonal versus Musashi-1 (1:100, Abcam^®^, Waltham, MA, USA), mouse monoclonal protein versus nestin (1:800; #MAB353—Merck Millipore^®^, Billerica, MA, USA), and rabbit polyclonal versus Sox-2 (1:2000; #Ab97959—Abcam^®^, Waltham, MA, USA).

Three wash steps were performed in 0.1 M PBS solution. Incubation with the second antibodies, which were Alexa Fluor A488 or A555 (1:1000, #A11001, #A11008—Thermo-Fisher^®^, Grand Island, NE, USA), was performed with 5 μg/mL DAPI (1:5000, D9542, Sigma-Aldrich^®^, Merck^®^, St. Louis, MO, USA) for 1 h in a 1% solution of BSA and 0.1 M PBS. Three washing steps were again performed in 0.1 M PBS solution. The mounting of the glass coverslips was performed on slides containing Mowiol^®^ (4-88, Sigma-Aldrich^®^, Merck^®^, St. Louis, MO, USA). The slides were stored at 5 °C in the dark.

### 2.6. Histological Sectioning and Immunohistochemistry

After preparation, the MGB was fixed in 4% paraformaldehyde (PFA, Sigma-Aldrich^®^, Merck^®^, St. Louis, MO, USA) for 1 h. Incubation of the fixed tissue was performed in ascending order with 10%, 20%, and 30% sucrose for 24 h each. The tissue was then cryoprotected in Tissue-Tek O.C.T. (Sakura^®^ Finetek Europe, Umkirch, Germany) and frozen in liquid nitrogen. The cryostat (CM1510S, Leica^®^, Wetzlar, Germany) was used to cut the slides into 9 µm sections. The sections were carefully mounted on Superfrost slides (Hartenstein^®^, Würzburg, Germany). Subsequently, the sections were postfixed in 4% PFA for 5 min and blocked in 10% BSA in 0.3% Triton X-100 (Sig-ma-Aldrich^®^, Merck^®^, St. Louis, MO, USA) for 1 h after three TBS-T washing steps. For immunohistochemistry, sections were incubated with the following primary antibodies in 1% BSA solution in 0. 3% Triton X-100 for 24 h: mouse monoclonal antibody to ß-tubulin (1:500, Sigma Aldrich^®^, Merck^®^, St. Louis, MO, USA), rabbit polyclonal antibody to ATOH1 (1:100, Santa Cruz^®^, Dallas, TX, USA), or rabbit polyclonal antibody to Sox-2 (1:100, Abcam^®^, Waltham, MA, USA). After incubation with the primary antibodies, three wash steps were performed with TBS-T. Incubation with the secondary antibodies coupled to Alexa 488 or Alexa 555 (1:800, 1:500, Invitrogen^®^, Carlsbad, CA, USA) and 5 μg/mL DAPI (1:5000, Sigma-Aldrich^®^, Merck^®^, St. Louis, MO, USA) followed. The samples were rinsed again in triplicate with TBS-T. Embedding was performed in Mowiol^®^ (4-88, Sigma-Aldrich^®^, Merck^®^, St. Louis, MO, USA).

### 2.7. Cytomorphometric Analysis and Digital Images

Digital images of cultures and preparations were acquired using a Leica^®^ DMI-8 fluorescence microscope and Leica Application Suite X software v3.0.1 (Leica^®^, Wetzlar, Germany). Neurospheres and histological sections were analyzed using a confocal laser scanning microscope (Fluoview FV3000, Olympus^®^, Shinjuku, Tokyo, Japan). Culture dishes were scanned by transmitted light technique using a 5× objective in tile scan mode. This allowed quantification of the neurospheres. The acquired image files were analyzed using Fiji/ImageJ V2.0.0 software [18]. The coverslips containing immunocytochemically stained preparations were also scanned in tile scan mode using a 20× objective. This allowed careful analysis to be performed subsequently. Final image composition was performed using Ado-be^®^ InDesign CC 2023 v18.1 software (Adobe Inc., San Jose, CA, USA).

### 2.8. Statistical Analysis

All collected data was compiled using Microsoft^®^ Excel 2023 V16.70 (Microsoft Corporation, Redmond, WA, USA) spreadsheets and statistically analyzed with GraphPad^®^ Prism 9.5.0 software (Graphpad Software Inc., San Diego, CA, USA). First, a column analysis (Shapiro-Wilk normality test) was performed to determine whether a Gaussian normal distribution of the data was present. Subsequently, data were analyzed using the ordinary one-way ANOVA test followed by a Tukey multiple comparison test. A *p*-value < 0.05 was considered to be statistically significant. Reproducible results were obtained from six or more samples.

## 3. Results

### 3.1. Increasing Neurosphere Size and Number over Time Demonstrate the Proliferation Capacity of MGB NSCs

**Figure 2:**
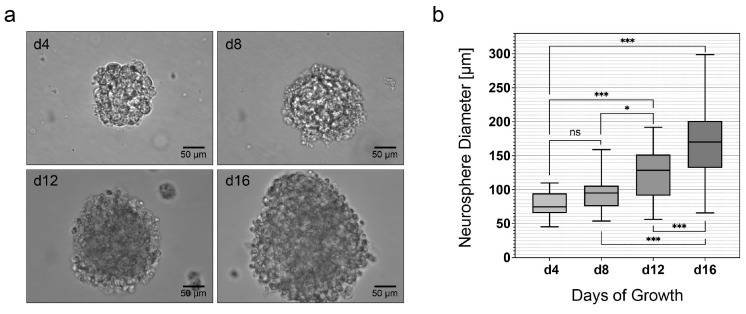
Highlighting the proliferation capacity of the MGB NSCs by the increase in the diameter of neurospheres in the NSC medium. (**a**) Formation of primary neurospheres from neural stem cells of the MGB of the PND 8 rat after 4, 8, 12, and 16 days in cell culture with NSC medium. (**b**) Diameter of primary neurospheres over time up to day 16 in cell culture. Boxplots show the median with the upper and lower quartiles, and whiskers mark the upper and lower maximum values; asterisks indicate the level of significance: ns = non-significant, * *p* < 0.05, *** *p* < 0.001.

Spherical and freely floating neurospheres developed in the cell cultures of dissociated cells from the MGB after four days. The diameter of these primary neurospheres increased consistently over time (Figure 2a). Between adjacent measurement time points, diameters increased 119% (n.s.) from d4 (78.51 ± 17.31 µm) to d8 (93.62 ± 21.35 µm), 131% (*p* = 0.0142) from d8 to d12 (122.9 ± 38.23 µm), and 143% (*p* < 0.0001) from d12 to d16 (175.2 ± 55.01 µm). Furthermore, an increase of 157% (*p* < 0.0001) was observed between d4 and d12 and 143% (*p* < 0.0001) between d8 and d16. There was an overall increase of 223% (*p* < 0.0001) between d4 and d16 (mean ± SEM; *n* = 30 neurospheres) (Figure 2b). 

**Figure 3:**
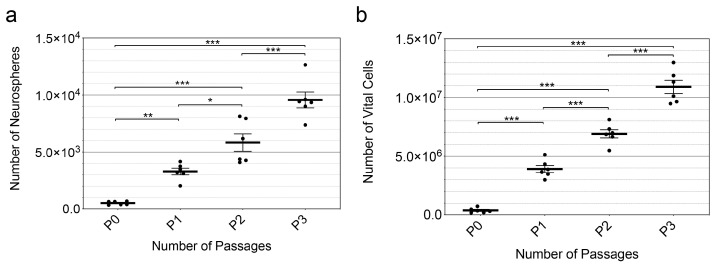
The significant increase in neurospheres and single cell numbers across multiple passages demonstrate the theoretically immortal mitotic self-renewal of NSCs. (**a**) The number of neurospheres per organ formed increased from P0 to P3. (**b**) The total number of cells within the neurosphere cultures continuously increased significantly via the passages P0-P3. The central horizontal bars show the mean; error bars depict the Standard Error of the Mean (SEM); each dot represents a cell culture, *n* = 6; asterisks indicate the level of significance, * *p* < 0.05, ** *p* < 0.005, *** *p* < 0.001.

After 30 days, the passage of cell cultures was performed, and the formation of secondary, tertiary, and quaternary neurospheres was analyzed. For this purpose, the number of neurospheres and the number of vital cells in the culture per animal were analyzed (*n* = 6). 524.3 ± 63.2 primary neurospheres per culture/animal or 10.7 ± 1.3 primary neurospheres per 1000 viable cells were formed. The number of neurospheres in culture increased steadily with time and across passages. On average, the number of neurospheres increased from P0 (524 ± 154) to P1 (3294 ± 723) by 629% (*p* = 0.0083), from P1 to P2 (5830 ± 1871) by 177% (*p* = 0.0164) and from P2 to P3 (9567 ± 1713) by 164% (*p* = 0.0005). Furthermore, an increase of 1113% was observed between P0 and P2 (*p* < 0.0001) and 290% between P1 and P3 (*p* < 0.0001). Overall, the number of spheres increased by 1826% (*p* < 0.0001) from P0 to P3; (mean ± SEM; *n* = 6 cell cultures) (Figure 3a). 

Single-cell analysis similarly revealed a continuous increase in the number of neurospheres across passages. On average, the number of vital single cells increased from P0 (377,500 ± 207,744) to P1 (3,904,500 ± 740,006) by 1034% (*p* < 0.0001) from P1 to P2 (6,904,500 ± 866,262) by 177% (*p* < 0.0001) and from P2 to P3 (10,904,500 ± 1,389,967) by 158% (*p* < 0.0001). Furthermore, the transition from P0 and P2 (*p* < 0.0001) resulted in an increase of 1829% and the transition from P1 and P3 in a rise of 279% (*p* < 0.0001). Overall, the number of vital cells increased by 2889% (*p* < 0.0001) from P0 to P3; (mean ± SEM; *n* = 6 cell cultures) (Figure 3b).

### 3.2. Neural Stem Cell Markers Are Expressed in Cells of the MGB In Vitro and In Vivo

**Figure 4:**
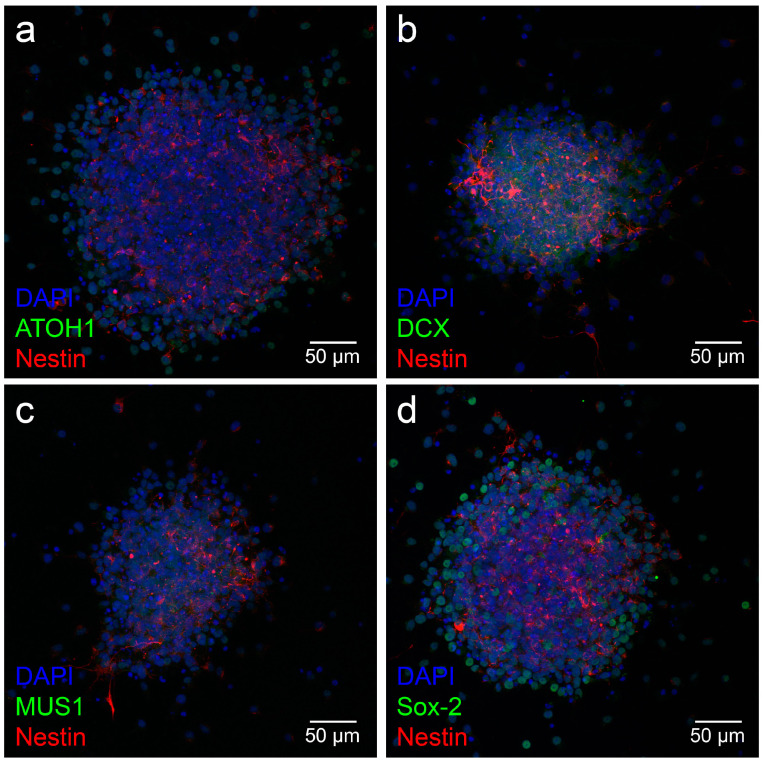
The expression of neural stem cell markers in neurospheres from cells of the PND 8 MGB. (**a**–**d**) Cells inside the neurospheres and cells emigrating from the spheres were stained positively for the neural progenitor cell marker nestin (red); cell nuclei were stained blue with DAPI. (**a**) The nuclei of cells inside the sphere and its branches showed positive labeling for the transcription factor ATOH1 (green). (**b**) The cytoplasm of the out-migrating cells and those inside the neurospheres were stained with the neural migration protein DCX (green). (**c**) The neural stem cell marker Musashi-1 (green) was positive in the cytoplasm of cells within the neurospheres and their branches. (**d**) The nuclei in the neurospheres and their branches showed positive labeling for the transcription factor Sox-2 (green).

Neurospheres were stained with the neural stem cell and progenitor markers doublecortin (DCX), Sox-2, Musashi-1, ATOH1 and nestin for further analysis. Expression of the transcription factors ATOH1 and Sox-2 showed colocalization with DAPI in the nuclei. The neuronal migration protein DCX and the RNA-binding protein Musashi-1 were detected in the cytoplasm of the neurospheres. In addition, cells within the neurospheres of all ages showed positive labeling of the progenitor marker nestin in their cytoplasm (Figure 4). 

**Figure 5:**
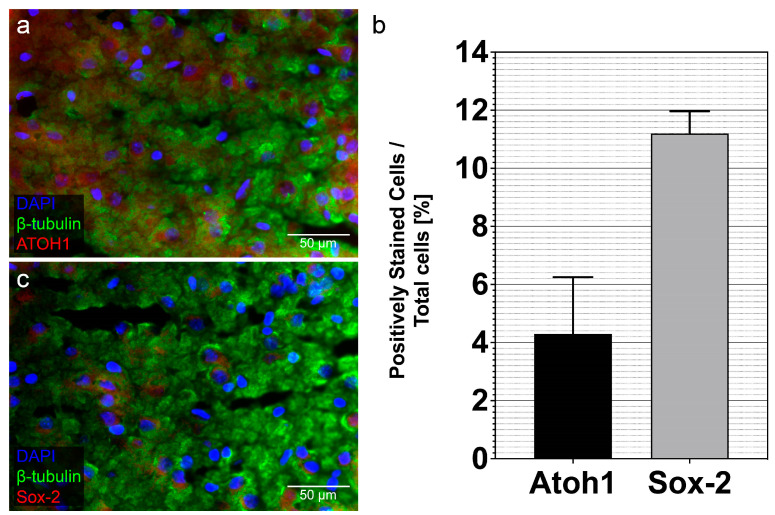
Neural stem cell markers are detectable in in vivo tissue sections of the MGB. (**a**) The transcription factor ATOH1 (red) is expressed in the nucleus of cells, and the cytoplasm of cells was positive for ß-tubulin (green). Cell nuclei are stained blue by DAPI. (**c**) The transcription factor Sox-2 (red) is expressed in the nucleus of cells. β-tubulin (red) is expressed in the cytoplasm of the cells. Cell nuclei are stained blue by DAPI. (**b**) Results of the immunohistochemical analysis of the neural stem cell markers ATOH1 and Sox-2 in histological sections of the PND 8 rat MBG. The proportions of positively stained cells in relation to the total number of cells on coverslips were evaluated. The bar charts show the mean and Standard Error of the Mean (SEM).

The neural stem cell and progenitor markers ATOH1 and Sox-2 were also detected in histological sections of the MGB. ATOH1 and Sox-2 were detected in the nucleus in colocalization with DAPI (Figure 5a,c). For a more detailed analysis, a systematic examination of stem cell marker-positive stained cells in relation to DAPI-positive cells was performed (Figure 5b). A total of 45,110 cells were counted and analyzed. Overall, a proportion of 4.29 ± 1.95% stained positive for ATOH1 and 11.19 ± 0.76% for Sox-2 (mean ± SEM; *n* = 3 histological sections).

### 3.3. MGB NSCs Differentiate into All Cell Types of the Neuroectodermal Cell Lineage

**Figure 6:**
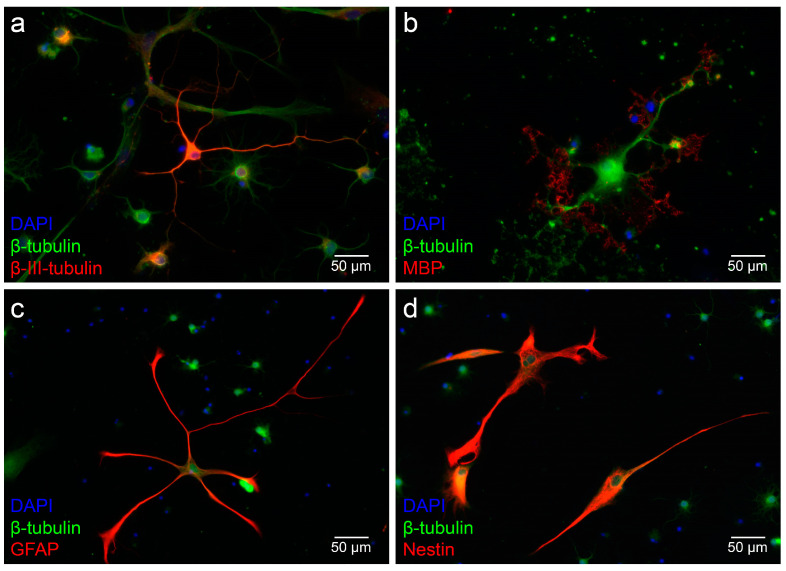
NSCs of the MGB differentiate into all cells of the neuroectodermal lineage after 6 days on glass coverslips in the differentiation medium (DIF). (**a**) β-III-tubulin (red) labels neuronally differentiated cells. The β-III-tubulin-positive cells had neuron-typical spindle-shaped and slender somata. (**b**) Oligodendrocytes showed positive labeling of myelin basic protein (MBP, red). MBP staining shows labeling and peripheral onset myelination. (**c**) Astrocytes were identified by glial fibrillary acidic protein (GFAP, red) staining. These cells displayed the star-shaped morphology typical of astrocytes. (**d**) Undifferentiated progenitor cells were stained with nestin (red). (**a**–**d**) The cytoskeleton of all viable cells was stained with β-tubulin (green). The cell nuclei were stained with DAPI (blue).

Single cells from dissociated neurospheres of the MGB developed into progenitor cells, neurons, and neuroglia. The neuron-specific marker β-III-tubulin was detected in axons and the cytoplasm of neurons. MBP plays a crucial role in the myelination of nerves and therefore represents the myelination processes of oligodendrocytes. The staining of GFAP identified astrocytes. Similarly, the progenitor cell marker nestin was used to detect neural progenitor cells from the dissociated single cells of the neurospheres from the MGB (Figure 6).

**Figure 7:**
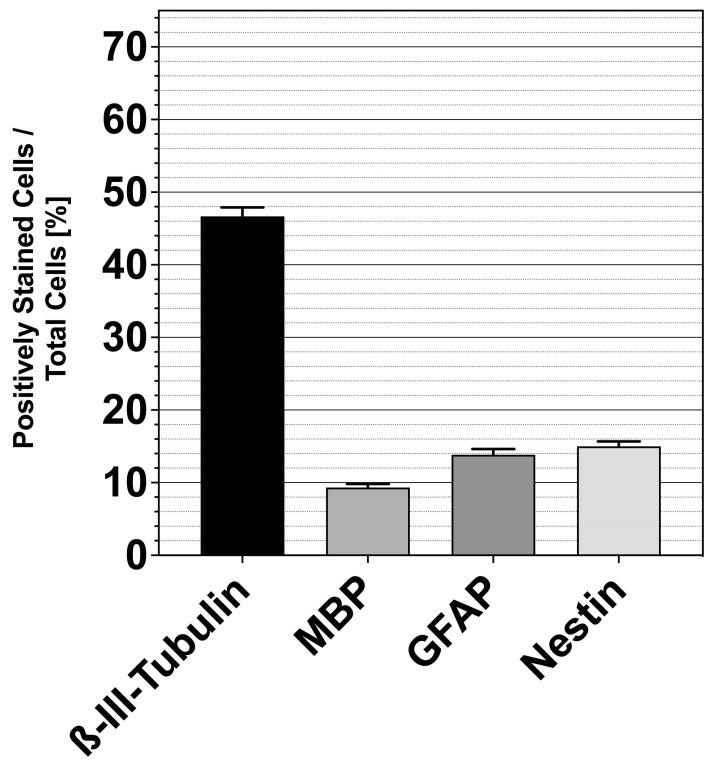
A large proportion of differentiating MGB NSCs develops a proneural fate. Results of the immunocytochemical analysis of the plated single cells of the PND 8 rat MBG after 6 days on glass coverslips in differentiation medium (DIF). The proportions of positively stained cells in relation to the total number of cells on coverslips were evaluated. The bar charts show the mean and Standard Error of the Mean (SEM).

The number of specifically labeled single cells was determined in relation to β-tubulin-positive cells. A total of 19,547 cells were counted. The percentage of neuron-specific β-III-tubulin-positive cells to β-tubulin-positive cells was 46.67 ± 1.13%. GFAP-specific evaluation yielded a rate of 13.83 ± 0.79% for cells of astrocytic lineage. Regarding the assessment of MBP, a percentage of 9.33 ± 0.49% resulted for oligodendrocytes. A portion of 15.00 ± 0.68% for the neural progenitor cell marker nestin was obtained (mean ± SEM; *n* = 6 cell cultures) (Figure 7).

## 4. Discussion

It has been possible to isolate NSCs in vitro from the MGB of PND 8 Sprague-Dawley rats, culture them as neurosphere assays and multiply and passage them over several weeks. Similarly, immunocytological and immunohistological methods were used to detect neural stem cell and neural progenitor markers. Dissociated single cells derived from the neurospheres formed all neuroectodermal cell lineage cell types in differentiation assays. These properties—mitotic and unlimited self-renewal, formation of progenitor cells, and multipotent differentiation into all neuroectodermal cell lineage cell types—are the cardinal criteria of neural stem cells [3]. These cardinal criteria were demonstrated for the first time in the MGB of the postnatal rat and indicated strong evidence that NSCs are present in the MGB of the postnatal rat. Due to the lack of a direct detection method of NSCs, indirect detection of NSCs of the MBG was performed. This approach has been recommended by several authors [2].

### 4.1. The Proliferation Capacity of the MGB NSCs Is Comparable to Other Neural Stem Cell Niches of the Auditory Pathway

A cell culture system designed explicitly for NSCs is used for the in vitro studies, a serum-free cell culture medium as described above (2.2). For this purpose, fibroblast growth factor FGF-2 and epidermal growth factor EGF are used, whose decisive influence on the microenvironment of NSCs has already been described in the literature [19]. The proliferative and inhibitory effects on the differentiation of FGF-2 and EGF are explicitly necessary and used in in vitro experiments to detect neural stem cells, and the withdrawal of growth factors induces cell differentiation [2]. Adding FGF-2 and EGF allows stimulation of the previously quiescent stem cell niche and offers the investigation and analysis of its proliferative capacity. Due to closely related activation patterns between neural stem cells and brain tumors, this interaction must occur in a physiologically controlled manner [20]. Previous studies on neural stem cells in the auditory system emphasized those results and demonstrated that no relevant number of neurospheres could be cultured in cell cultures lacking FGF-2 and EGF to demonstrate the cardinal properties of neural stem cells [21]. Free-floating neurospheres formed in the cell cultures, which are a correlate for mitotic self-renewal and proliferation of NSCs, as a small proportion of the cells in the neurospheres are NSCs [22].

Continuously enlarging and proliferating neurospheres formed in MGB cell cultures from PND 8 Sprague-Dawley rats. Analysis of the size of the neurospheres allows an assessment of NSCs for neurosphere formation. With increasing neurosphere size, increased proliferative capacity can be assumed [23]. An analysis of the diameters of the neurospheres within the first 16 days in culture was performed. This showed a significant increase in the diameter of the neurospheres over time. After 4 days in culture, the average diameter of the neurospheres was 78.51 ± 17.31 µm (mean ± SEM; *n* = 30 neurospheres) (Figure 2b). In a study in which the prosencephalon of rat fetuses was harvested on embryonic day 14.5 and examined on day four in culture for the diameter of the neurospheres formed, it was found that under comparable conditions (37 °C/5% CO_2_), the majority of the neurospheres were between 50 and 100 µm in size. Thus, the neurospheres of the postnatal MGB had similar diameters of neurospheres after four days in culture-like neurospheres of the embryonic rat prosencephalon [24]. The maximum average diameter recorded was 175.2 ± 10.2 on the 16th day in culture (Figure 2a). These results were compared with the literature. Nearly identical studies were performed along the auditory pathway in the IC of PND 6 Sprague-Dawley rats. At day 16 in culture, an average neurosphere diameter of 267.8 ± 60.4 μm was observed [13]. Thus, larger average diameters of neurospheres were present in the IC of PND 6 animals. Neural stem cell potential analysis of mouse auditory cortex revealed an average diameter of 165.2 ± 13.9 µm in tertiary neurospheres from 3-day-old mice and a diameter of 86.6 ± 6.0 µm in 14-day-old mice after 15 days in culture [14]. With limited comparability, because diameters of tertiary rather than primary neurospheres were recorded and different animals were compared at different ages, very similar diameters were present in the auditory cortex and the MGB after 15 and 16 days in culture, respectively.

In cell cultures from the MGB of PND 8 Sprague-Dawley rats, 524.3 ± 63.2 primary neurospheres were formed per culture/animal or 10.7 ± 1.3 primary neurospheres per 1000 viable cells (approximately 1%) (mean ± SEM; *n* = 6 cell cultures) (Figure 3a). The number of primary neurospheres in relation to the number of cells studied and per culture/animal of the MGB was compared with results in the literature. In the IC of PND 6 rats, a comparable ratio of approximately 1588 ± 606 neurospheres per culture/animal or 8.2 ± 3.1 neurospheres/1000 viable cells (0.8%) was found under the same conditions [13]. Since the study conditions are almost identical, except for the age of the animals, this provides an excellent opportunity to compare the respective primary neurosphere formation. In the CN of PND 6 rats, the formation of 1.4 ± 0.4 spheres/1000 cells (0.14 ± 0.04%) was observed after three weeks in culture [21]. In another study of the CN of PND 3 mice, a more pronounced neurosphere formation of about 3 spheres/100 viable cells (approximately 3%) was described [25]. A comparatively lower ratio was found in the fourth ventricle of adult mice, with about 0.2 ± 0.06 spheres/1000 viable cells after eight days [26]. Because of the different approaches, comparing the results of the other studies is difficult. The cell culture medium (DMEM vs. Neurobasal^®^ medium) and the mode of passage (enzymatic vs. mechanical; after 8 days vs. after 30 days) have a decisive influence on the proliferative capacity [27]. Additionally, the species studied (rat vs. mouse) is a significant factor affecting the outcome of the studies [28].

Further passage analyses were performed to investigate whether there was unlimited potential for mitotic division and self-renewal. After 30 days each, passage occurred, and primary, secondary, tertiary, and quaternary neurospheres formed. Analysis of the number of neurospheres and the number of vital cells within the passages revealed a constant significant increase with the increasing passage. Thus, the cells of the MGB of 8-day-old Sprague-Dawley rats exhibit the capacity for unlimited mitotic self-renewal, a cardinal feature of neural stem cells [29].

### 4.2. The Expression Patterns of NSC Markers in the MBG Show Localization-Specific Differences

Another cardinal criterion of NSCs is the formation of neural progenitor cells. Therefore, the question arose whether neuronal stem cell and progenitor markers can be detected in the cultivated neurospheres from cells of the MGB of PND 8 rats. Supplementary histological sections were examined for neural progenitor and stem cell markers. 

The neural progenitor and stem cell markers ATOH1, nestin, Musashi-1, doublecortin (DCX) and Sox-2 were examined. The transcription factor ATOH1 belongs to the basic helix-loop-helix (bHLH) family of transcription factors and provides a proneural fate to the cell [30]. It contributes to the development of the central auditory pathway [31]. Nestin is an intermediate filament expressed in developing neuroepithelial cells and is detectable until the cells reach terminal differentiation [32]. Sox-2 is an HMG box transcription factor that plays a critical role in the maintenance of multipotency as well as the ability to self-renew [33]. Musashi-1 is an RNA-binding protein that plays a significant role in neurogenesis and influences the fate of neuronal precursor cells [34]. In addition, the neurospheres were examined for DCX, a neuronal migration protein found in young and migrating neurons [35]. The expression of all neuronal progenitor and stem cell markers examined were detected in the neurospheres of PND 8 rats. The markers were detectable not only in the cells of the neurospheres but also in the emigrating cells (Figure 4a–d). These cells could be neural progenitor cells that can grow out in a monolayer [36]. Nestin could be detected within the neurospheres, as well as in the cells growing out of them (Figure 4a–d). Expression of Sox-2 and ATOH1 was restricted to the nucleus and occurred in cells within the neurospheres and in cells outgrowing them (Figure 4a,d). In contrast, Musashi-1 was expressed mainly perinuclearly but also intranuclearly (Figure 4c) [37]. DCX-positive cells could be detected in the cytoskeleton of the cells (Figure 4b). It is known that DCX is a microtubule-associated protein, and colocalization with nestin is characteristic of proneural progenitor cells [38]. These markers’ expression and growth patterns in neurospheres are consistent with findings from previous studies.

Since the neuronal progenitor and stem cell markers Sox-2 and ATOH1 were detected in neurospheres derived from cells of the MGB, the question arose whether these stem cell markers can also be seen in histological sections of the MGB. ATOH1 exhibited co-staining with DAPI, known from former studies (Figure 5a) [39]. Similarly, Sox-2 positive cells could be detected in the histological sections of the MGB (Figure 5c). These results are consistent with the literature [40]. To evaluate the expression capacity of these markers, a quantitative evaluation of marker-positive cells was performed in relation to DAPI-positive cells. A proportion of 4.29 ± 1.95% stained positive for ATOH1 and 11.19 ± 0.76% for Sox-2 (mean ± SEM; *n* = 3 histological sections) (Figure 5b) was found. There are only a few comparable studies in the literature. Despite different evaluation methodologies (marker-positive cells per µm3 vs. marker-positive cells per DAPI-positive cells), a similar trend was observed in the CN of PND 9 rats. There are approximately twice as many Sox-2 positive cells as ATOH1 positive cells [12]. Analysis of ATOH1 and Sox-2 in dissociated single cells from neurospheres of the IC of PND 6 rats revealed 3.11 ± 2.62% expression for ATOH1 and 2.3 ± 3.23% expression for Sox-2 in regard to all cells plated out [13]. Thus, the expression capacity of ATOH1 is comparable to the results obtained from tissue sections of the MGB. Interestingly, significantly lower expression of Sox-2 was present in the single-cell studies of the IC. In contrast, very similar expressions of the two markers were detected in neurospheres of the IC of PND 6 rats [41]. PND 8 animals were not evaluated in this study, but there was already a significant decrease in ATOH1 compared with PND 12 animals. Thus, lower ATOH1 expression in neurospheres can be assumed in PND 8 animals. In this regard, in a previous study, the expression level of Sox-2 of different neural progenitor populations in the telencephalon was examined. Interestingly, Sox-2 was found to be expressed differently by other cell types. The differences in the expression of Sox-2 between the MGB and the IC are likely due to Sox-2 being expressed by different cell populations [42].

Furthermore, Sox-2 was expressed prominently in postmitotic differentiated neurons of the lateral geniculate body. This is an adjacent nucleus of the MGB in the visual thalamus, whose projection neurons to the visual cortex showed strong Sox-2 expression. These results were unexpected, as Sox-2 is known to maintain stem cell capacity and is generally not detected in differentiated neurons. Thus, it could be an exception in projection neurons of the thalamus, which would explain the increased expression of Sox-2 in the MGB [43]. Interestingly, the role of Sox-2, especially in the sensory system, was emphasized in differentiated cells. In addition to its importance as a neural stem cell marker, Sox-2 performs key functions in both neurons and glial cells [44]. Since the thalamus has a significant role in sensory perception, the results of this work indicate a localization-specific distinct expression of Sox-2.

Neuronal progenitor and stem cell markers could be detected in cells of the MGB in both in vitro and in vivo studies. Cells of the MGB possess the ability to form neural progenitor cells, which is another cardinal feature of NSCs.

### 4.3. NSCs of the MGB Show a Proneural Differentiation Fate 

Neurobasal cell medium was used for the differentiation experiments by adding B27 and retinoic acid and a deprivation of growth factors. The importance of retinoids in vitro for cell differentiation, analogous to an essential role in central nervous system maturation in vivo, has been described previously [45]. Plating of dissociated cells from neurospheres in a DIF medium demonstrated the differentiation of neurons, astrocytes, and oligodendrocytes. 

ß-III-tubulin is a neuronal marker commonly used to show the ability to differentiate in neurons. Neuronally differentiated cells formed a network with neighboring cells (Figure 6a). These morphological and immunocytological features were also demonstrated in similar experiments with NSCs [46]. In addition, ß-tubulin-positive cells with a typical spoke structure with a prominent nucleus were detected, and their morphology is characteristic of oligodendrocytes. Myelin basic protein (MBP) was seen in the peripheral processes of these glial cells, identifying them as oligodendrocytes (Figure 6b) [47]. These are also found in vivo adjacent to neuronal cells, forming the myelin sheaths around axons and playing an essential metabolic role [48]. Astrocytes could be unambiguously identified by GFAP, which is the classical marker for staining differentiated astrocytes (Figure 6c) [49]. Similarly, cells could be positively identified for nestin. These cells were still in a precursor stage and could be identified morphologically and immunocytologically as neural progenitor cells (Figure 6d) [50]. Furthermore, the marker-positive single cells were related to all β-tubulin-positive cells to analyze the capacity for differentiation (Figure 7). The percentage of neuron-specific β-III-tubulin-positive cells was 46.67 ± 1.13%. The GFAP-specific evaluation revealed a portion of 13.83 ± 0.79%, in MBP a rate of 9.33 ± 0.49% for oligodendrocytes. A percentage of 15.00 ± 0.68% was obtained for the neural progenitor cell marker nestin (mean ± SEM; *n* = 6 cell cultures). Comparison with the differentiation capacity of the IC of PND 6 rats revealed both similarities and crucial differences. In the IC, 50.97 ± 2.22% ß-III tubulin-positive cells were present, yielding similar results to the MGB. The percentage of MBP-positive cells was 14.36 ± 1.14%, which was higher in the IC compared with the MGB. The most significant difference was in GFAP-positive cells. The IC showed 5.06 ± 1.12% astrocytic cells. Nestin-positive cells had a very similar expression profile of 13.24 ± 6.07% [41]. Interestingly, the MGB assumes a critical role in tinnitus pathology, and GFAP expression has been shown to remain stable in the rat tinnitus model compared to the auditory cortex [51]. The pronounced expression of GFAP in the MGB may indicate the stable expression at even early ages.

Dissociated single cells from neurospheres of the MGB of PND 8 rats differentiated into all neuroectodermal cell lineage cell types following the withdrawal of growth factors in the DIF medium. The ability to differentiate into all cell types of the neuroectodermal cell lineage is another cardinal feature of NSCs [36].

### 4.4. Limitations of the Study

There may be some limitations within the present study. Neural stem cells play a crucial role in neurogenesis. In this study, neural stem cells were cultured in vitro under the influence of highly proliferative growth factors. As a result, dormant neural stem cell niches are activated, and the distinction between an active and mitotically dormant stem cell niche is more difficult. As it became apparent in this study, the differentiation fates of neural stem cells differ. Thus, understanding the heterogeneity of neural stem cells and their physiological regulation is limited. Inferences about the mechanisms that activate quiescent stem cell niches and regulate progenitor cells for neurons and glial cells are hampered by the model used in this study. Insight into these mechanisms is provided by non-mammalian vertebrates, such as zebrafish. This is because, for example, Drosophila NSCs transit synchronously between a quiescent and proliferative state. This facilitates differentiation between stages. In rodents, active and quiescent NSC niches coexist [52]. However, the cardinal features of neural stem cells can be detected using the rodent model, which was the aim of this study, and it is a well-studied model. Another weakness of this work is that it focused on early postnatal animals. However, an assessment of neural stem cell potential with increasing and into adult age would be of interest. As it is known, early postnatal animals have a significantly higher stem cell potential. This has enabled sufficient propagation and systemic analysis. The results of this work should serve as a basis for further investigations.

## 5. Conclusions

In the present study, NSCs were identified in the neonatal MGB. Cells were characterized in vitro and in vivo. The isolated cells exhibited the cardinal features of NSCs. Compared with other auditory pathway nuclei in which NSCs have been detected, a comparable neural stem cell potential was present in the postnatal rat MGB.

In addition, some neurogenic factors with specific influence on neurogenesis are known. In the future, it may be possible to influence neural tissue regeneration specifically. Potential therapeutic approaches to brain or brainstem damage due to injury, toxins, or ischemia could be developed by targeted modulation of neurogenesis. However, a detailed understanding of cellular and molecular processes is necessary to regulate neurogenesis therapeutically. Therefore, the existence of NSCs in the MGB contributes to understanding the auditory pathway’s developmental features.

## Data Availability

The data used to support the findings of this study are included within the article.
